# Supplementary material for: In-Depth Bioinformatic Analyses of Nidovirales Including Human SARS-CoV-2, SARS-CoV, MERS-CoV Viruses Suggest Important Roles of Non-canonical Nucleic Acid Structures in Their Lifecycles
Source: Front Microbiol. 2020 Jul 3;11:1583. doi: 10.3389/fmicb.2020.01583 (PMC7347907; doi:10.3389/fmicb.2020.01583)
Supplement: Supplementary file 1 [file Data_Sheet_1.zip › SM_3_IR_analyses_description.docx]

| 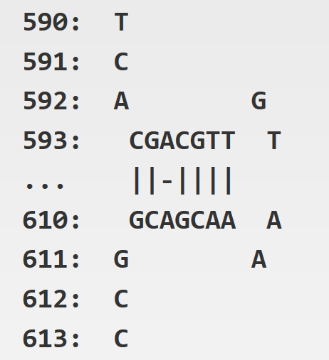 |
| --- |
| Supplementary Figure S1: Visualisation of inverted repeat. This IR has following parameters: size 7, spacer size 4, mismatch 1 (7-4-1). |
|  |
| **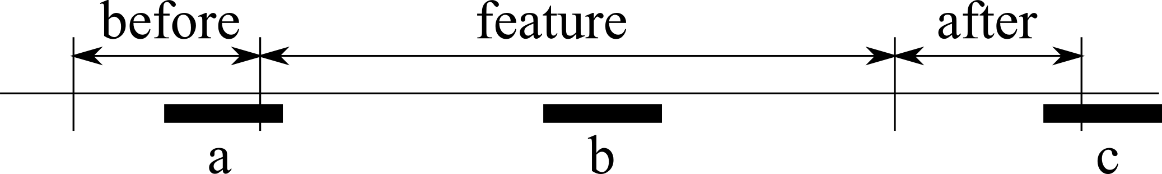** |
| Supplementary Figure S2: Neighbourhood of an annotated feature. Example of possible IR occurrence around features and its classification: a) An inverted repeat is considered to be in near neighbourhood because it overlaps only partially with a feature. b) An inverted repeat overlapping fully with a feature and therefore is considered to be inside. c) An inverted repeat is not considered to be in near neighbourhood because it is not fully overlapping neither with a feature or its neighbourhood. |
